# Complex sympathetic arousal during negative emotional stress

**DOI:** 10.1192/j.eurpsy.2021.1299

**Published:** 2021-08-13

**Authors:** Z. Visnovcova, N. Ferencova, L. Bona Olexova, I. Tonhajzerova

**Affiliations:** 1 Biomedical Center Martin, Jessenius Faculty of Medicine in MartinComenius University in Bratislava, Martin, Slovak Republic; 2 Department Of Physiology, Jessenius Faculty of Medicine Comenius University, Martin, Slovak Republic

**Keywords:** sympathetic nervous system, electrodermal activity, heart rate variability, negative emotional stress

## Abstract

**Introduction:**

The autonomic nervous system (ANS) plays a key role in maintenance of the homeostasis and adaptability of the body to different stimuli. The disturbances of ANS, especially sympathetic dysregulation in stress response, are associated with various disorders.

**Objectives:**

Thus, we aimed to study the sympathetic arousal in response to negative emotional stress and during recovery using heart rate variability (HRV) nonlinear analysis (symbolic dynamics parameter 0V%) and skin conductance level (SCL) as sympathetically-mediated indices in healthy students.

**Methods:**

Seventy students (age: 23.1±0.2yr., 39 females) were examined during complex stress response: baseline – negative emotional stress – recovery. RR intervals (for HRV analysis) and electrodermal activity were continuously recorded during each period lasting six minutes. Evaluated parameters: HRV nonlinear analysis - symbolic dynamics index 0V% as cardiac sympathetic index, skin conductance level (SCL) as sympathetic cholinergic index.

**Results:**

Regarding electrodermal activity, the parameter SCL significantly increased in response to negative emotional stress (p<0.001) and remained higher after stress (recovery phase, p<0.001). Symbolic dynamics index 0V% was without significant changes.

**Conclusions:**

Our findings revealed increased sympathetically-mediated index SCL in response to negative emotional stress and in recovery phase indicating higher sympathetic arousal during complex stress response in young people. Surprisingly, cardiac sympathetic index 0V% was not sensitive to detect discrete changes in sympathetic arousal to negative emotion. We suggest that detailed knowledge about complex sympathetic regulatory mechanisms to emotional stress in healthy probands represents the first step for understanding of pathomechanisms leading to abnormal stress response in mental disorders.

**Conflict of interest:**

This study was funded by the Slovak Scientific Grant Agency under grants VEGA 1/0044/18; VEGA 1/0190/20 and Ministry of Health of the Slovak Republic under the project registration number 2018/20-UKMT-16.

